# The effect of a multicomponent exercise protocol (VIVIFRAIL©) on inflammatory profile and physical performance of older adults with different frailty status: study protocol for a randomized controlled trial

**DOI:** 10.1186/s12877-021-02030-2

**Published:** 2021-01-29

**Authors:** Marina Petrella, Ivan Aprahamian, Ronei Luciano Mamoni, Carla Fernanda de Vasconcellos Romanini, Natália Almeida Lima, Everson de Cássio Robello, Daniele Lima da Costa, Vinicius Nakajima An, Bianca Nobre Aguirre, Júlia Riccetto Galdeano, Isabela Cunha Fernandes, Salma S. Soleman Hernandez, Matteo Cesari, John E. Morley, Mikel Izquierdo, Richard C. Oude Voshaar

**Affiliations:** 1 Group of Investigation on Multimorbidity and Mental Health in Aging (GIMMA), Division of Geriatrics, Department of Internal Medicine, Faculty of Medicine of Jundiaí, 250 Francisco Telles st, Jundiaí, 13202-550 Brazil; 2 Department of Psychiatry, University Medical Center Groningen, University of Groningen, Groningen, The Netherlands; 3 Geriatric Unit, Fondazione IRCCS Ca’ Granda Ospedale Maggiore Policlinico, University of Milan, Milan, Italy; 4 Division of Geriatrics, St louis University Medical School, St Louis, MO USA; 5 Navarrabiomed, Complejo Hospitalario de Navarra (CHN)-Universidad Pública de Navarra (UPNA), IdiSNA, Pamplona, Spain; 6 CIBER of Frailty and Healthy Aging (CIBERFES), Instituto de Salud Carlos III, Madrid, Spain; 7 Grupo GICAEDS, Programa de Cultura Física, Deporte y Recreación, Universidad Santo Tomás, 110311 Bogotá, Colombia

**Keywords:** Clinical trial, Elderly, Frailty, Inflammation, Physical exercise

## Abstract

**Background:**

To investigate whether an exercise intervention using the VIVIFRAIL© protocol has benefits for inflammatory and functional parameters in different frailty status.

**Methods/design:**

This is a randomized clinical trial in an outpatient geriatrics clinic including older adults ≥60 years. For each frailty state (frail, pre-frail and robust), forty-four volunteers will be randomly allocated to the control group (*n* = 22) and the intervention group (*n* = 22) for 12 weeks. In the control group, participants will have meetings of health education while those in the intervention group will be part of a multicomponent exercise program (VIVIFRAIL©) performed five times a week (two times supervised and 3 times of home-based exercises). The primary outcome is a change in the inflammatory profile (a reduction in inflammatory interleukins [IL-6, TNF- α, IL1beta, IL-17, IL-22, CXCL-8, and IL-27] or an increase in anti-inflammatory mediators [IL-10, IL1RA, IL-4]). Secondary outcomes are change in physical performance using the Short Physical Performance Battery, handgrip strength, fatigue, gait speed, dual-task gait speed, depressive symptoms, FRAIL-BR and SARC-F scores, and quality of life at the 12-week period of intervention and after 3 months of follow-up.

**Discussion:**

We expect a reduction in inflammatory interleukins or an increase in anti-inflammatory mediators in those who performed the VIVIFRAIL© protocol. The results of the study will imply in a better knowledge about the effect of a low-cost intervention that could be easily replicated in outpatient care for the prevention and treatment of frailty, especially regarding the inflammatory and anti-inflammatory pathways involved in its pathophysiology.

**Trial registration:**

Brazilian Registry of Clinical Trials (RBR-9n5jbw; 01/24/2020). Registred January 2020. http://www.ensaiosclinicos.gov.br/rg/RBR-9n5jbw/.

## Background

Frailty is a biological syndrome characterized by a reduction in physiological reserves and a decrease in resistance to stressors [1, 2]. The worldwide prevalence of frailty is 4–16% over 65 years old and 25% in those aged 85 or older [3, 4]. Frailty is characterized as a main cause of death in seniors [5] and significantly increases the risk of falls, disability, hospitalizations and long-term care [6]. According to the theoretical model of the frailty phenotype, a multisystemic impairment of homeostasis trigger a cascade of dysregulation during the aging process, causing a cycle of energy reserve reduction [1, 2, 7]. Physical frailty is the biological model most used for diagnosis and is composed by three or more among five components (unintentional weight loss, self-reported fatigue, low handgrip strength, low physical activity; and low walking speed). Pre-frailty is identified by one or two of these components [2].

Chronic inflammation is a key element in its pathophysiology [7] and has its origin through factors that include aging changes, genetic and metabolic variations, environmental stressors, lifestyle, and chronic and acute diseases. Frail individuals have higher levels of inflammatory mediators, such as C-reactive protein (CRP), tumor necrosis factor alpha (TNF-α) and interleukin-6 (IL-6) directly influencing frailty onset by degrading proteins, or indirectly, affecting the functioning of metabolic pathways [8, 9]. Low-grade inflammation is associated with decreased strength and muscle mass, its intensity is inversely proportional to the practice of muscle strengthening activity and can be reduced with resistance training [10, 11].

Few controversial studies investigated the effect of exercise on inflammatory biomarkers in frailty. A stabilization of IL-6 and CRP was observed in frail and pre-frail older persons during physical training and nutritional support, while controls presented an increase in these cytokines [12]. Moreover, those who improved physical performance lowered IL-6 levels. In another study, a resistance training for knee flexors and extensors muscles did not influence low-grade inflammation in older adults. However, plasma levels of TNF type 1 receptors were inversely related to the muscle strength at the end of 12-weeks [13]. In youngers, the increase in the serum level of TNF-α after intervention correlated with reductions in muscle mass after a 4-week follow-up period, suggesting that TNF-α may play a role in muscle loss due to detraining [14]. Collectively, these studies show the potential anti-inflammatory role of physical activity.

Thus, this single-center parallel randomized controlled trial will allow to investigate whether a low-cost multicomponent exercise program called VIVIFRAIL© [15, 16] has benefits for reducing inflammation and improve the functional status among older persons with different frailty status. This program is performed according to the functional capacity and has been demonstrated to be a safe and effective to reverse the functional decline associated with acute hospitalization in seniors [17, 18]. The specific objectives of this RCT will be evaluate the effectiveness of the VIVIFRAIL© [15, 19] program with respect to the concentration of inflammatory and anti-inflammatory mediators and in the functional status of older persons in the different frailty status during 12-weeks. It is hypothesized that older persons submitted to the VIVIFRAIL© exercise protocol will present a decrease in serum levels of pro-inflammatory interleukins, associated with an increase in serum levels of anti-inflammatory interleukins and, secondarily, an improvement in the functional status is expected.

## Methods/design

### Design and participants

The present study is a single-center parallel randomized controlled trial comparing a multicomponent physical activity (i.e. VIVIFRAIL©) to usual health promotion. Participants are older adults from the Geriatrics Outpatient Center of Jundiaí Medical School, State of São Paulo, southwester of Brazil. These patients usually come from primary care centers or access the geriatric outpatient care directly.

**Fig. 1:**
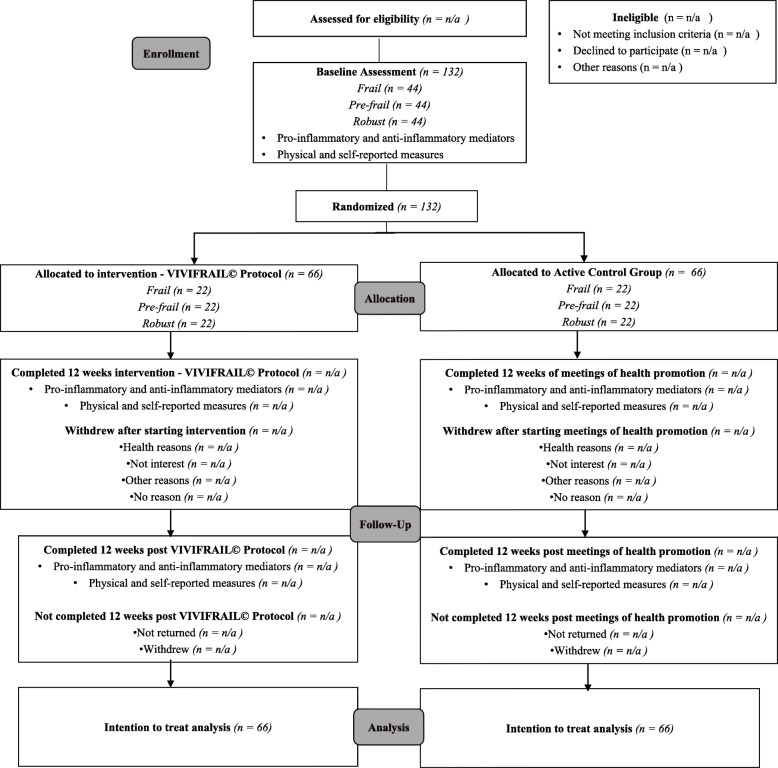
CONSORT Fluxogram showing the study design and the flow of participants

Volunteers who meet the inclusion criteria will be randomly allocated to the intervention or control groups by an independent person, who is blind to their clinical data or frailty status. The data for participants in both groups will be obtained at three different times: at the beginning of the study, at the end of the study and after 3 months after the end of the intervention. The study flowchart is illustrated in Fig. 1. This study protocol follows the standard protocol for clinical trials in accordance with the SPIRIT2013 statement and follows the CONSORT statement for clinical trial transparency [20, 21]. The study is registered on the Brazilian Registry of Clinical Trials (REBEC) platform under the license RBR-9n5jbw. The study was approved by the Research Ethics Committee of the Jundiaí Medical School. For any ancillary study that data collection/request is not covered in the original informed consent process for this main trial, another signed consent will be obtained from every participant.

### Recruitment and inclusion criteria

The initial recruitment will be carried out with all older adults who followed up at the clinic during the year of 2019. The older adults that are receiving care at the clinic, that are aged 60 years or older, and who have signed the informed consent will be included in the study. The exclusion criteria will be: probable cognitive impairment identified using the 10-point cognitive screening instrument (10-CS) with a final score of 0 to 5 points; any contra-indication for the VIVIFRAIL© [19] (Table 1), life-expectancy less than 12 months, permanent or temporary inability to walk; localized loss of strength and aphasia due to severe stroke; severe impairment of motor skills, speech or affectivity associated with Parkinson’s disease in an advanced/unstable stage; severe hearing or vision deficits; cancer and autoimmune diseases in activity; cancer in the last 3 years; inability to perform the Short Physical Performance Battery (SPPB); limb amputation; and physical activity or physical therapy in the last 6 months.

**Table 1:** Absolute and relative contraindications for physical activity according to VIVIFRAIL©Protocol

VIVIFRAIL© Contraindications	
Absolute	• Acute heart attack (recent 3–6 months) or unstable angina; • uncontrolled atrial or ventricular arrhythmias; • aortic dissecting aneurysm; • severe aortic stenosis; • acute endocarditis / pericarditis; • uncontrolled high blood pressure (> 180/100 mmHg); • acute thromboembolism; • acute or severe heart failure; • acute or severe respiratory failure; • uncontrolled postural hypotension; • uncontrolled acute decompensated diabetes mellitus or low blood sugar; • a recent fracture in the last month (strength training); • any other circumstance the doctors believes prevents doing physical activity
Relative	• A recent fracture in the last 3 months (strength training) • Infections that affect one’s general condition • A pathology that causes serious functional limitation (Barthel scale of less than 20)

Those who meet the inclusion criteria and agree with the study criteria presented by the researcher carrying out the recruitment (a medical doctor or physical therapist) will sign the consent information term and will be submitted to the assessment of the Frailty Physical Phenotype criteria proposed by Fried and colleagues (2001), according to the description below:

- Fatigue: it will be evaluated through self-report using questions 7 and 20 of the Center for Epidemiological Studies - Depression (CES-D) scale. The volunteers will be asked about the frequency in which they perceived to make an effort to perform usual tasks and the frequency with which they were unable to carry out their activities in the last week. Positive affirmation “most of the time” or “always” in any of the two questions will be considered a manifestation of fatigue.

- Unintentional weight loss in the last year: older person with unintentional weight loss greater than 5% of body weight will be scored according to this criterion, adjusted for sex and body mass index (BMI).

- Hand grip strength will be performed using a manual hydraulic dynamometer (Jamar hand dynamometer, model J00105, Lafayette Instrument Company, Lafayette, Lousiania, USA). This instrument is composed of a closed hydraulic system that measures the amount of force produced by an isometric contraction applied over its loops, and the hand grip is recorded in kilogram-force. It will be performed with the individual sitting in a chair without armrests and with their feet resting on the floor, with adducted shoulder, the elbow flexed at 90°, the forearm in neutral position and the wrist between 0 and 30° in extension. Prior to the evaluation, the volunteer will be familiarized with the instrument for the opportunity to handle the dynamometer before recording the measurements and will be guided on the evaluation procedure. The volunteer will be instructed to tighten the dynamometer handle tightly held by the volunteer’s dominant hand and verbal commands will be provided to encourage volunteers to produce maximum grip strength. Three evaluations will be carried out, with a one-minute rest between each one and the average of the repetitions, in kilogram-force (kgf), will be used for statistical analysis. Low grip strength will be indicated by the average values ​​interpreted according to sex and BMI. For females, will be considered low handgrip strength values ≤17 kg for those with BMI ≤ 23 kg / m^2^, values ≤17.3 kg for a BMI between 23.1–26 kg / m^2^, values ≤18 kg for a BMI between 26.1–29 kg / m^2^ and values ≤21 kg for females with BMI > 29. For males, will be considered a low hand grip strength values ≤29 kg for those with BMI ≤ 24 kg / m^2^, values ≤30 kg for a BMI between 24, 1–26 kg / m^2^, values ≤30 kg for a BMI between 26.1–28 kg / m^2^ and values ≤32 kg for males with BMI > 28.

- Low gait speed: it will be assessed when asking the older person to walk at their usual speed for 4.6 m, repeating the route 3 consecutive times. The average speed obtained in each of the routes will be interpreted according to the height and gender of the participants. Will be considered with low gait speed the male participants with a height ≤ 173 cm who took a time ≥ 7 s or those with height > 173 cm who took a time ≥ 6 s. For females’ participants, will be considered with low gait speed those higher than 159 cm who took ≥7 s or those higher than 159 cm who took a time ≥ 6 s to complete the test.

- Low level of physical activity: Low physical activity is defined as spending less than 383 cal per day for males and 270 cal for females, corresponding to the lowest quintile of energy expenditure in the older population. The energy expenditure will be measured with some items of the Minnesota Leisure Activity Questionnaire, which has been translated and validated in Portuguese [22]. Based on self-report frequency and duration of physical activities (including sports and leisure activities), the calculation of caloric expenditure will be performed by multiplying the metabolic equivalent of the task (MET) of each activity by the body weight (Kg), time (minute) and the predetermined measure of 0.0175.

After evaluating the Frailty Physical Phenotype criteria, participants who have 1–2 of these components will be considered as pre-frail, 3 or more components as frail, and those without any as robust.

### Randomization and blinding

Participants will be allocated to groups (intervention or control group) randomly in 1:1 rate. The randomization sequence will be generated by an independent professional using an online system (www.randomization.com) to allocate 22 participants from each frailty group (i.e. frail, pre-frail and robust) to the control group (*n* = 66) and in the intervention group (*n* = 66). The type of intervention that the participant will undergo during the study will be indicated on a sheet of paper inserted in opaque, sequentially numbered, sealed envelopes. These envelopes will be prepared by a researcher who is not involved in enrolling the participants, in assigning them to their groups or performing follow-up measurements and will be delivered after baseline measurement. Follow-up measurement at the end of intervention period and after 3 months will be performed by a researcher who will be unaware of group allocation.

The professionals responsible for the physical and blood assessments will be blind to participant’s group. It will not be possible to blind the allocation of groups from the professional responsible for the intervention since the VIVIFRAIL protocol change according to patients’ profile. It will not be possible to hide the intervention of the participants, who will know about the allocation in different groups at random. However, they will not be aware of which group they were allocated. Raters and data-analysts will keep blinded in relation to the intervention performed by the volunteer.

### Measurements

Outcome measures will be done at three moments: baseline, after 12 weeks (end of intervention), and 24 weeks.

#### Inflammation

The peripheral blood of all participants will be collected. From everyone, approximately 9 mL of blood will be collected in tubes containing clot activator and 9 mL in tubes containing sodium heparin for peripheral blood mononuclear cells (PBMCs) and polymorphonuclear cells (PMNs) isolation. The serum will be separated after clot formation by centrifugation at 1200 *g* for 15 min at room temperature, then the serum will be collected, aliquoted and immediately stored at 20 °C. The cytokine measurement will be performed using ELISA with specific commercial kits for IL-6, TNF- α, IL1beta, IL-17, IL-22, CXCL-8, IL1RA, and IL-27 (R&D Systems and Biolegend manufacturers’ instructions).

PBMCs will be isolated using Ficoll-Hypaque density gradient centrifugation (density 1.077 - Pharmacia Biotech, Piscataway, NJ, USA). After removing PBMCs, the buff-coat cells (containing PMNs) will be collected and diluted in fresh RPMI medium and submitted to new centrifugation over a Ficoll-Hypaque solution at a density of 1.119). After separation, PBMCs and PMNs will be resuspended (2 × 10^6^ cells/mL) in RPMI medium supplemented with fetal bovine serum (10%), L-glutamine, sodium pyruvate and gentamicin (RPMI-S) and distributed in 24-well plates. Cultures will be maintained without additional stimulation or stimulated with ultrapure LPS (100 ng/mL - Invivogen) and PMA (100 ng/mL - Sigma) for PMNs and with ultrapure LPS (100 ng/mL - Invivogen) and PHA (10 mg/mL) for PBMCs. The cells will be kept for 24 h, at 37 °C in a CO_2_ incubator (5%). After the culture period, the supernatant will be collected and stored at − 80 °C and will be evaluated by ELISA with commercial kits for each cytokine (IL-10, IL-4, IFN-gamma, IL-17, TNF- α, IL-6) (R&D Systems and Biolegend).

The details of the pro-inflammatory and anti-inflammatory mediators investigated in this study are presented in the Table 2.

**Table 2:** Pro-inflammatory and anti-inflammatory mediators

Mediator	Major Cellular Sources	Major Activities
**IFN-gamma**	Th1 cells, NK cells	Activation of inflammatory macrophages (M1), inhibition of Th2 responses, induction of leukocyte migration.
**IL-1beta**	Monocyte/macrophages, PMNs.	Synthesis of acute-phase proteins by hepatocytes, induction of local and systemic inflammatory effects.
**IL-1RA**	Monocyte/macrophages, dendritic cells.	Specific inhibitor of IL-1alpha and IL-1beta by competition with the cellular receptor.
**IL-4**	Th2 cells, mast cells, B cells	Induction of Th2 lymphocyte development, inhibition of pro-inflammatory and Th1 cytokine production, activation of anti-inflammatory macrophages (M2).
**IL-6**	Monocyte/macrophages, T cells, PMNs.	Produced during the inflammatory response, induces the synthesis of acute-phase proteins by hepatocytes, but presents an inhibitory effect on TNF- α and IL-1 production by macrophages.
**IL-8 (CXCL8)**	Monocytes/macrophages, PMNs.	Major chemotactic cytokine (chemokine) for neutrophils.
**IL-10**	Monocyte/macrophage, Th2 cells, B cells	Inhibition of pro-inflammatory cytokine production by macrophages and neutrophils, inhibition of Th1 cytokine production.
**IL-17**	Th17 cells	Activation of neutrophils, induction of extracellular matrix remodeling.
**IL-22**	Th17 and Th22 cells	Induction of epithelial cell proliferation and production of antimicrobial peptides by epithelial cells and neutrophils.
**IL-27**	Macrophages, dendritic cells.	Antagonist of Th1, Th2 and Th17 inflammatory responses, induction of Tr1 (regulatory) cells.
**TNF-** α	Monocyte/macrophages, PMNs, T cells.	Synthesis of acute-phase proteins by hepatocytes, recruitment, and activation of cells into inflammatory sites, induction of insulin resistance.

#### Physical performance

The SPPB battery allows the evaluation of the performance of the lower limbs. It is composed of the assessment of standing balance, gait speed and muscle strength. The SPPB varies between 0 and 12 points, with higher score representing better performance. It is a valid instrument for screening for frailty and predicts disability, institutionalization and death, so that a change of 1 point represents a relevant issue [23, 24].

#### Secondary measures

The gait evaluation during the simultaneous performance of a cognitive task is a useful tool for functional evaluation in frail older patients, allowing to predict falls [25]. The participants will perform a 6 m gait velocity test (6 m-GVT) while naming animals aloud. The cognitive score during the dual task will be measured by counting the number of animals named. Depression symptoms will be evaluated throughout the Geriatric Depressive Scale (GDS) [26], composed by 15 items in which mental health related questions are asked in reference to how the participants felt over the past week. The quality of life will be evaluated though the EQ-5D questionnaire (“EuroQol - a new facility for the measurement of health-related quality of life,” 1990). The questionnaire its composed by five dimensions (mobility, self-care, usual activities, pain/discomfort and anxiety/depression with 3 levels (no problems, some problems, and extreme problems). All participants also will be assessed with FRAIL-BR [1] and SARC-F [27, 28].

To register the physical activity frequency after the intervention period, a diary will be used and the times per week of physical activity performed by the participants will be computed.

### Interventions

#### Control group

To ensure that possible changes in the serum levels of biomarkers were not related to frailty, participants allocated to control group will participate in monthly meetings of health promotion. To minimize bias effect of socialization on the perception of health, the meetings will include lectures on topics related to health, such as food, use of medicines, prevention and health promotion and socialization activities [29]. Participants will be guided to continue the usual primary and secondary health care (public or private) and community activities. Telephone contact will be made after absences from meetings.

#### Intervention group

The VIVIFRAIL© [15, 19] multicomponent exercise intervention protocol was developed in Europe (Erasmus+programme, European Union) and consists of resistance training, gait retraining, and balance training. Different functional capacity levels will be determined based on the scores obtained from the SPPB and the 6-m gait velocity test, with each leading to the recommendation of a certain customized multicomponent physical exercise program (Program A, B, C1, C2 or D). These programs are composed by arm and leg strength and power exercises, balance and coordination to prevent falls, flexibility and cardiovascular endurance exercises [15, 19].

After the initial assessment and categorization of the volunteer in any of the physical exercise programs, a multicomponent program will be carried out for 12 weeks, totaling 60 sessions. Two weekly supervision sessions will be held, lasting 60 min each, with an interval of 1 day between them. Participants will receive the materials (elastic bands, towel and bottles with water/sandy) to perform the exercises and will be trained to perform exercises individually at home with a frequency of 3 days a week, in order to complete 5 days of activity per week. Instructions will be properly adapted to the focused population and will be provided to the volunteers in the Portuguese language.

**Fig. 2:**
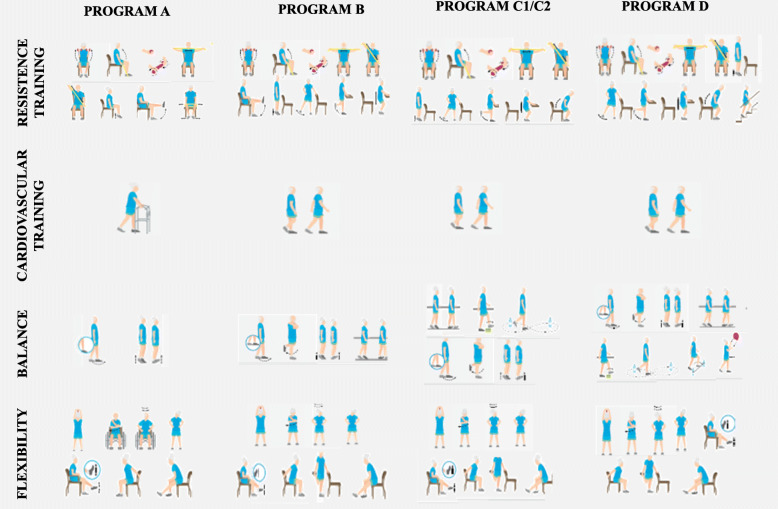
Proposed exercises illustration of each program of the VIVIFRAIL© multicomponent exercise methodology. Modified from Izquierdo and colleagues (2017)

The VIVIFRAIL© program is composed by arm and leg strength and power exercises, balance and coordination to prevent falls, flexibility and cardiovascular endurance (i.e walking) exercises [16, 30]. The proposed exercises’ sets and repetitions, intensity of progression of each Program are shown in Table 3 and illustrated in Fig. 2.

**Table 3:** Proposed exercises sets, repetitions (Reps) and intensity (Intst) of progression of each program of the VIVIFRAIL© multicomponent exercise methodology

		Program A	Program B		Program C 1 and C2	Program D
**Resistance training**	Sets / Reps	Week 1–2: 2 sets / 10 reps Week 3–4: 2 sets / 10 reps Week 5–6: 2 sets / 10 reps Week 7–8 2 sets / 10 reps Week 9–10: 2 sets / 10 reps Week 11–12: 2 sets / 10 reps	Week 1–2: 2 sets / 10 reps Week 3–4: 2 sets / 12–15 reps Week 5–6: 3 sets / 12 reps Week 7–8 2 sets / 10 reps Week 9–10: 2 sets / 12–15 reps Week 11–12: 3 sets / 12–15 reps			
	Intst	Determine the weight that allows to do the exercise properly without stopping yet makes feel as though they have made an effort by the end: Week 1–6: 30 times Week 6–12: 20 times				
**Cardiovascular training**	Sets / Reps	Time walking / rest / times Week 1–4: 5–10s / 10s / 5–7 Week 5–6: 10–15 s / 20s / 5–7 Week 7–8: 15–30s /20s / 5–10 Week 9–10: 30–45 s / 20s / 5–10 Week 11–12: 45–60s /20s/12–15	Time walking / rest / times Week 1–4: 20s / 10s / 5–7 Week 5–6: 20–25 s / 20s / 5–7 Week 7–8: 25–35 s /20s / 5 Week 9–10: 45 s / 20s / 5 Week 11–12: 50–70s /20s/12–15	Time walking / rest / times		Time walking / rest / times Week 1–2: 15 min / 30s / 2 Week 3–4: 15 min / 30s / 3 Week 5–6: 20 min / 30s / 2 + 15 min Week 7–8: 30-35 min /30s / 2 Week 9–10: 50–70 min Week 11–12: 50–70 min
				C1	Week 1–2: 3-5 min / 30s / 2 Week 3–4: 5 min / 30s / 2 Week 5–6: 5 min / 30s / 3 Week 7–8: 5-7 min /30s / 3 Week 9–10: 7–12 min Week 11–12: 12–20 min	
				C2	Week 1–2: 8-10 min / 30s / 2 Week 3–4: 10 min / 30s / 2 Week 5–6: 5-10 min / 30s / 3 Week 7–8: 15-25 min Week 9–10: 25–30 min Week 11–12: 30–40 min	
	Intst	Usual walking pace				
**Balance training**	Sets / Reps	Remain in the same position, and do 2–3 times with each leg, counting to: Week 1–2: 5–10 Week 3–4: 10 Week 5–6: 15 Week 7–8: 20 Week 9–10: 25 Week 11–12: 30	Remain in the same position and do 2–3 times with each leg, counting to: Week 1–2: 10 Week 3–4: 15 Week 5–6: 20 Week 7–12: 30 + Walk 10 steps/ 2 times + Walk in a relaxed way and step over 5 obstacles / 8 times	Remain in the same position counting to: Week 1–2: 10 Week 3–4: 15 Week 5–6: 20 Week 7–12: 30 These exercises will be done 2 times with each leg, followed by: Walk 10 steps/ 2 times + Walk in a relaxed way and step over 5 obstacles / 8 times		
**Flexibility**	Intst	-Change the position of the arms; -Cross your arms - Use of different surfaces - Eyes closed.				
	Sets / Reps	Week 1–6: 2 sets of 3 reps Week 7–12: 3 sets of 3 reps				
	Intst	Remain in the same position for 10–12 s

The intervention will be discontinued due to any condition or adverse event that does not allow the performance of exercises with security or if requested by any participant. If necessary, one medical team will be disposable to offer assistance at any moment.

The participants will be instructed to does not enroll in other physical activity intervention during the period of 12 weeks of intervention.

After initial assessment and categorization, the multicomponent program will be carried out for 12 weeks, totaling 60 sessions. Two weekly supervisioned sessions will be held (60 min) with an interval of 1 day between them. Participants will receive all materials (elastic bands, towel and bottles with water/sand) to perform the exercises and will be trained to perform exercises individually at home 3 days weekly in order to complete 5 days per week. Instructions will be adapted to the focused population and will be provided in Portuguese. Adherence to the intervention will be monitored using a frequency control spreadsheet before the start of each session. Participants will be instructed to report possible adverse events.

Participants may withdraw from the study for any reason at any time. The investigator also may withdraw participants from the study in order to protect their safety and/or if they are unwilling or unable to comply with required study procedures. To promote participant retention and complete follow-up, all the participants will receive written feedback regarding the assessments performed.

### Outcomes

Primary and secondary outcomes will be accessed at baseline, after 12 weeks (end of intervention) and 24 weeks (Table 4).

**Table 4:** Primary and secondary outcomes for the participants of the study

	Measurement	Baseline (T0)	Post 12 week of Intervention (T1)	12 weeks of follow-up (T2)
***Primary outcomes***	Plasma levels of inflammatory cytokines	x	x	x
	Function of leukocytes in the peripheral circulation	x	x	x
***Secondary outcomes***	Short Physical Performance Battery	x	x	x
	Maximal isometric handgrip strength (kg.f)	x	x	x
	Gait speed (m/s)	x	x	x
	Fatigue	x	x	x
	Geriatric depression Scale (GDS)	x	x	x
	Dual-task_gait + verbal_ (m/s)	x	x	x
	Quality of Life (EQ-5D)	x	x	x
	FRAIL-BR	x	x	x
	SARC-F	x	x	x
	Physical activity frequency during follow-up			x

#### Primary outcomes

The primary outcome measure is a change in the inflammatory profile after a 12-week multicomponent physical exercise program (VIVIFRAIL©), whether a reduction in inflammatory mediators in serum (IL-6, TNF- α, IL1beta, IL-17, IL-22, and CXCL-8) or culture supernatants (IL-6, TNF- α, IFN-gamma and IL-17) or an increased production of anti-inflammatory mediators (IL1RA and, IL-27) in serum or IL-10 and IL-4 in culture supernatants. We hypothesize that the intervention would lead to an anti-inflammatory profile.

#### Secondary outcomes

Secondary outcomes are a change in: (1) physical performance according to the SPPB, (2) handgrip strength, (3) fatigue according to specific items of the CES-D scale, (4) gait speed, (5) dual-task gait speed, (6) depressive symptoms, (8) FRAIL and SARC-F scores, and (7) quality of life.

We hypothesize that the intervention group could present at least a 1-point increase in the SPPB, better measurements of strength and gait speed, less referred fatigue and depressive symptoms, and better quality of life.

All the participants will be coded using numbers and the data will be stored electronically. After storage, data will be checked by a second researcher to ensure data quality*.* All participant information will be stored in areas with limited access. All records that contain names or other personal identifiers will be stored separately from study records identified by code number.

### Statistical analyses

First, sample calculation was based in a previously published results from a clinical trial of resistive physical intervention among frail older adults [31]. This study is similar to our design and outcomes. In this trial, outcomes involved inflammatory biomarkers. To date, VIVIFRAIL trials were done in inpatients and nursing home patients and without inflammatory biomarkers as outcome measurement. For a 30% reduction in IL-6 with 80% power and a 5% error, we would have 18 older adults in each group for 12 weeks. Assuming a drop out until 25% within this period, the groups should be composed of 22 elder adults each.

Statistical analyzes will be performed using the SPSS program (version 23.0; SPSS, Inc., USA) and R version 3.6.2. Initially, the Shapiro Wilk and Levene tests will be used to test the distribution and homogeneity of the data. Descriptive analysis will be performed and the means and standard deviation of the variables with normal distribution will be presented, and the median and maximum and minimum values ​​for the non-normal variables and 95% confidence intervals.

Analysis by intention to treat will be performed. A sensitivity analysis will be performed for missing data, through multiple imputation. To compare the variables between the groups, the Student t-test will be used or the Mann-Whitney test for non-parametric variables. To assess the difference between the moments of intervention, one-way analysis of variance (ANOVA-oneway) with repeated measures or the Friedman test for nonparametric variables will be used.

## Discussion

Immune dysregulation is underlying frailty, interacting with neuroendocrine dysregulation and neuromuscular dysfunction. Considered a marker of aging, chronic low-grade inflammation is associated with common diseases in aging, with adverse outcomes such as death and the functional impairment [2, 6, 32], in addition to being a possible pathophysiological mechanism of frailty (Gale, Baylis, Cooper, & Sayer, 2013). As it is a dynamic condition, an older person can move between the different stages of frailty status [29]. Therefore, investigating whether conservative interventions used for the prevention and treatment of frailty influence low-grade chronic inflammation in frail and pre-frail older persons will help to understand the physiological mechanisms of frailty.

In addition to improving essential aspects for maintaining the health and functionality of older persons (eg, increasing muscle strength), improving balance and preventing falls, physical exercise has been shown to decrease the levels of inflammatory biomarkers [33]. In a recent systematic review, the combination of muscle strengthening, and protein supplementation proved to be an effective and feasible intervention to reverse or prevent frailty [34]. However, the quality of the studies still leaves doubts about this evidence.

Since the balance between inflammatory and anti-inflammatory biomarkers has been suggested as a determinant for the severity of conditions associated with aging [35], understanding the effect of exercise on the inflammatory profile of frail and pre-frail older persons will contribute to a better understanding of perspectives for the prevention and treatment of frailty and will represent an advance in the knowledge about the use of biomarkers for monitoring frailty.

This is the first study to perform the VIVIFRAIL© methodology in a sample of frail and pre-frail outpatient older adults from a middle-income country in order to verify its effect on the inflammatory profile and physical functional parameters. The VIVIFRAIL© exercise protocol is a low-cost intervention that could be easily replicated in the community (Basic Health Units, Community Centers, etc.) [15, 19]. If our results confirm our hypothesis, this study could contribute with an easy to implement methodology to be disseminated acting at the person end public health level after further multicenter trials with a higher number of participants.

In the next future this project offers the opportunity to test and disseminate “in the real life” a novel prescription exercise tool (VIVIFRAIL©) [16, 30] with regard to the modification of low-grade chronic inflammation and in physical functional parameters of frail and pre-frail older outpatients. It will yield direct information about the effects of a specific and individualized designed exercise program in those geriatric conditions. In the long run, such results will contribute to the better understanding of physiopathological mechanisms of physical exercise in the treatment of frailty. It is also expected that the results of the present study will assist in advancing the use of inflammatory biomarkers and physical functional measures to monitor chronic low-grade inflammation in frail and pre-frail older persons included in programs of prevention and rehabilitation of frailty. Another important expected result in our project is to stimulate the prescription of exercise in this population in order to prevent disability. Finally, with the actual target of a novel era of precision medicine, our results could be a significant step forward to a “precise prescription of exercise recipe” for older patients with frailty. Important questions remain about safety, effectiveness, and the inherent variability between people in response to exercise [36, 37].

## Data Availability

The datasets generated during and/or analyzed during the present study will be available from the corresponding author on reasonable request.

## Abbreviations

10-CS: 10-point cognitive screening instrument
6 m-GVT: 6 m gait velocity test
BMI: Body mass index
CES-D: Center for Epidemiological Studies - Depression
CRP: C-reactive protein
GDS: Geriatric Depressive Scale
IL: Interleukin
Kgf: Kilogram-force
MET: Metabolic equivalent of the task
PBMCs: Peripheral blood mononuclear cells
PMNs: Polymorphonuclear cells
REBEC: Brazilian Registry of Clinical Trials
SPPB: Short Physical Performance Battery
TNF-α: Tumor necrosis factor alpha
